# Overexpression of TLR2 and TLR9 on monocyte subsets of active rheumatoid arthritis patients contributes to enhance responsiveness to TLR agonists

**DOI:** 10.1186/s13075-015-0901-1

**Published:** 2016-01-13

**Authors:** Patricia Lacerte, Alexandre Brunet, Benoit Egarnes, Benjamin Duchêne, Jacques P. Brown, Jean Gosselin

**Affiliations:** Laboratory of Innate Immunology, Centre de recherche du CHU de Québec, Université Laval, Québec, QC Canada; Department of Molecular Medicine, Université Laval, Québec, QC Canada; Division of Rheumatology, Centre Hospitalier Universitaire de Québec, Université Laval (CHUL), Québec, QC Canada; Infectious and Immune Diseases, Centre de recherche du CHU de Québec-Université Laval (CHUL), Québec, QC Canada

**Keywords:** Monocytes subsets, TLR2, TLR9, Rheumatoid arthritis, Blood cells, Synovial monocytes, Viral agonists, Innate response

## Abstract

**Background:**

Synovial infiltration of monocytes is commonly associated with inflammation in rheumatoid arthritis (RA). Toll-like receptors (TLRs) are innate sensors that recognize cell debris and microbial components in host, a process contributing to maintain chronic inflammation in RA. We assessed the expression levels of TLR2 and TLR9 in monocyte subsets of active RA patients and characterized their cytokine profiles in response to synthetic and viral TLR2 and TLR9 agonists, including Epstein-Barr virus (EBV) which is suspected to contribute to RA symptoms.

**Methods:**

Prevalence of monocyte subsets CD14^++^ CD16^−^, CD14^+^ CD16^+^ and CD14^low^ CD16^++^ was evaluated in blood and synovial fluids of active RA patients and levels of TLR2 and TLR9 in monocyte subsets were measured by flow cytometry. Enriched monocytes derived from RA patients and healthy donors were stimulated *in vitro* with synthetic TLR2 and TLR9 agonists and with EBV particles or viral DNA. Intracellular cytokine profiles were determined in respective monocyte subsets. Finally, the presence of EBV genome was evaluated by real-time PCR in blood and synovial monocytes of RA patients.

**Results:**

Numbers of CD14^+^ CD16^+^ and CD14^low^ CD16^++^ were found to increase in blood of RA patients compared to healthy controls, while all three subsets were detected in synovial fluids. TLR2 is abundantly expressed on blood and synovial CD14^++^ CD16^−^ and CD14^+^ CD16^+^ monocytes from RA patients. Levels of TLR9 were increased on all three subsets of blood monocytes but markedly enhanced in monocytes isolated from synovial fluids. Compared to healthy controls, CD14^++^ CD16^−^ monocytes of RA patients displayed an enlarged capacity to produce proinflammatory cytokines after stimulation with synthetic TLR2 and TLR9 agonists while both CD14^++^ CD16^−^ and CD14^+^ CD16^+^ monocytes showed increased response to EBV stimulation. The presence of EBV genome was also detected in monocytes and neutrophils of a significant proportion of patients.

**Conclusion:**

Patients with active RA show an increased expression of TLR2 and TLR9 on monocyte subsets and display higher production of inflammatory cytokines in response to TLR agonists. The presence of EBV genome in monocytes and neutrophils reinforces the suspected role of the virus in the exacerbation of RA symptoms.

## Background

Rheumatoid arthritis (RA) is an autoimmune disease that is characterized by chronic joint inflammation that progressively leads to cartilage and bone destruction. It is generally believed that genetic factors are involved in the susceptibility to develop RA, but evidence also suggests that environmental factors, including viral infections, may have an influence on the incidence of disease. RA is characterized by synovial hyperplasia and inflammation resulting from a massive infiltration of inflammatory cells, including monocytes and neutrophils [1]. These cells play a key role in the progression of RA through the production of proinflammatory cytokines, leading to the development of an inflammatory environment and immune cell recruitment in the joints.

In humans, monocytes are a heterogeneous cell population composed of three distinct subsets based on their expression of CD14 and CD16 [2]. The CD14^++^ CD16^−^ classical subset, which is the most prominent of all circulating monocytes, is rapidly recruited to the sites of inflammation and appears to act as phagocytic scavenger cells and regulators of inflammation [3, 4]. The second monocyte subset expresses levels of both CD14 and CD16 (CD14^+^ CD16^+^). It is referred to as intermediate monocytes and is suggested to play a proinflammatory role, being increased in blood from patients with acute inflammation [5, 6]. The third subset comprises nonclassical monocytes that express low levels of CD14 and high levels of CD16 (CD14^low^ CD16^++^), which are often referred to as patrolling monocytes [7]. The two CD14^+^ subsets are thus recognized to expand in various inflammatory diseases and are suggested to play a significant role in disease processes [8, 9]. In RA patients, the frequency of monocytes expressing CD16 antigen (independently of the level of expression of CD16) was found to be increased in blood and synovial fluids [10, 11].

Toll-like receptors (TLRs) are expressed in all subsets of monocytes. These receptors function as detectors to recognize microbial motifs, and several debris and intracellular molecules released from necrotic cells [12, 13]. Triggering of TLRs by various stimuli leads to the activation of signaling pathways that culminate in the production of proinflammatory cytokines, a process driving aberrant and chronic inflammation in the joints of RA patients (reviewed in [14]). The role of TLRs in arthritis is underlined by the fact that resident and infiltrating cells in the inflamed joint were found to express various TLRs. While their functional roles are not yet clearly defined, different studies have attempted to identify which TLRs are associated with the severity of RA. For example, TLR3 and TLR4 have been found highly expressed in synovial fibroblasts from RA patients [15]. It was also suggested that RA patients carrying a TLR4 variant, Asp299Gly, have decreased susceptibility to RA [16]. Expression levels of TLR2 were found increased in CD14^+^ CD16^+^ blood monocytes isolated from RA patients compared to healthy controls [17] and such abundant expression of TLR2 was also detected in the synovial tissue of RA patients compared to patients with osteoarthritis [18, 19]. Monocytes and differentiated macrophages isolated from RA patients also have been found to have increased expression of TLR2 as compared to those from other forms of inflammatory arthritis [20]. While these observations suggest that TLR2 might contribute to sustaining inflammation in the joints, its role in the pathogenesis of RA remains to be clarified.

Little is known about the stimuli capable of triggering signals that result in synovial tissue damage. In addition to cell debris, viral infection has long been speculated to be a potential factor in RA. Several viruses have been suspected for many years to contribute to potentiate RA symptoms, including Epstein–Barr virus (EBV) [21, 22], cytomegalovirus (CMV) [23], parvovirus B19 [24] and hepatitis C virus [25], to name a few. EBV is still by far considered as a leading candidate to exacerbate autoimmune diseases, including RA [22, 26, 27]. Indeed, EBV has high prevalence in the population and persists in latently infected cells with continuous viral reactivation. This virus has also the potential to modulate the immune system and to infect monocytes and neutrophils [28, 29], two key cellular populations activated in RA. The results presented in this study demonstrate that monocyte subsets isolated from patients with active RA express elevated levels of both TLR2 and TLR9 and have enhanced responsiveness to synthetic and viral TLR2 and TLR9 agonists compared to controls. A significant fraction of blood monocytes and neutrophils from these patients were found to contain the EBV genome, supporting the possible contribution of this virus in the exacerbation of inflammation in susceptible RA patients.

## Methods

### Ethics statement

Experiments were performed in accordance with an internal review board-approved protocol at le CHU de Québec – Université Laval (Québec, Canada) (#105.05.06). All healthy donors and patients gave their written informed consent.

### Cohort recruitment

Twenty-three patients with active RA and long-standing disease (>4 years) were recruited for this study. Seven of them had a large synovial knee effusion when recruited. Clinical features of selected RA patients are presented in Table 1. When sampled, all patients had flare-up with high scores in the 28 swollen-joints count (14.4 ± 8.1) and most (16/23) had a high disease activity score in 28 joints-erythrocyte sedimentation rate (DAS 28-ESR) (≥5.1). Twelve EBV-seropositive healthy volunteers who did not experience inflammatory diseases and were not suffering from infectious diseases when recruited, are referred to as healthy controls. The presence of anti-EBV antibodies, e.g., anti-early antigen (EA), anti-Epstein–Barr nuclear antigen 1 (EBNA-1) and anti-viral capsid antigen (VCA), was tested in plasma of all RA patients and healthy controls by ELISA (DIASource Immunoassay, Louvain-La-Neuve, Belgium). Serologic results show that patients with active RA present abnormal elevated anti-EBV titers compared to seropositive healthy volunteers (data not shown).

**Table 1:** Clinical characteristic of selected patients

Characteristics	Values in patients with rheumatoid arthritis (n = 23)
Gender male/female, n	2/21
Age, years, mean ± SD	57.0 ± 10.8
Disease duration, y, mean ± SD	5.0 ± 7.3
Swollen joints, 0–28	14.4 ± 8.1
CRP, ± SD, mg/l	13.0 ± 12.7
ESR, ± SD, mm/h	19.4 ± 16.4
RF (+), n (%)	9 (39 %)
ACPA (+), n (%)	8 (35 %)
RF & ACPA (+) n (%)	7 (30 %)
Disease activity score in 28 joints-CRP	5.3 ± 1.5
DAS activity score in 28 joints-ESR	5.7 ± 1.5

Data are mean ± SD unless stated otherwise. *CRP* C-reactive protein, *ESR* erythrocyte sedimentation rate, *RF* rheumatoid factor, *ACPA* anti-citrullinated protein antibodies

### Isolation of monocytes and neutrophils from blood and synovial fluids

Plasma from healthy donors and RA patients and synovial fluids (SF) from RA patients were isolated by centrifugation. Mononuclear cells were isolated using Ficoll density gradient (Wisent, Québec, Canada) as reported [30], and were first separated from the lymphocyte population by cell adherence on Petri dishes. Monocyte subsets were analyzed by flow cytometry based on their expression of CD14 and CD16 using CD14-PE-Cy7 (M5E2) and CD16-A647 (3G8) antibodies (BD Biosciences, San Jose, CA, USA) (purity approximatley 99 %) (BD FACSAria, BD Biosciences). Classical, intermediate and nonclassical monocytes were specifically identified by selective gating strategy as follows: CD14^++^ CD16^−^ (classical), CD14^+^ CD16^+^ (intermediate) and CD14^low^ CD16^++^ (nonclassical). We found no evidence to indicate that blood dendritic cells (<1 %) contribute significantly to subpopulations of circulating monocytes [31]. Monocytes were enriched from synovial fluids with the same approach. Blood neutrophils from RA patients and healthy volunteers were isolated as previously described [28, 32]. Neutrophil purity (>98 %) was determined using intracellular staining with an anti-myeloperoxidase (MPO) antibody (5B8) (BD Biosciences). When indicated, the presence of cytokines (IL-1β, IL-6, TNFα, MCP-1) in synovial fluids from RA patients was determined using Cytometric Bead Array system (CBA FlexSet, BD Biosciences, San Jose, CA, USA).

### Flow cytometry analysis

Enriched monocyte subsets (identified as detailed above) and neutrophils were washed twice in Hanks Balanced Salt Sodium (HBSS; Wisent) and stained with TLR2-fluorescein isothiocyanate (FITC) (TL2.1) or TLR9-FITC (5G5) antibodies (Hycult Biotech, Uden, The Netherlands). Cells were fixed and permeabilized prior to TLR9 staining [30] and subsequently washed twice and resuspended in HBSS supplemented with 1 % bovine serum albumin (BSA; Wisent) for cytometry analysis. Expression of TLR2 and TLR9 was analyzed on each monocyte subset and neutrophils using a BD SORP LSR II and data analyzed with the BD FACS Diva (BD Biosciences) and FlowJo (FlowJo, LLC, Ashland, OR, USA) software.

### Cell treatment and intracellular cytokine staining

For intracellular cytokine staining (ICCS), peripheral blood mononuclear cells (PBMC) from healthy volunteers and RA patients were isolated using Ficoll density gradient. After an overnight resting, cells were washed and stimulated in vitro as indicated with EBV particles (multiplicity of infection of 1) or EBV DNA (40 μg/ml) [30, 33]. As positive TLR2 and TLR9 controls, cells were stimulated with lipoteichoic acid (LTA) (10 μg/ml) and type-B CpG-2006 (40 μg/ml), respectively. After a 5-hour stimulation in the presence of BD GolgiPlug™ (protein transport inhibitor) (BD Biosciences), stimulated cells were harvested and cytokine expression levels were assessed by intracellular flow cytometry [34]. Supernatants were sporadically tested for protein transport blockage and cytokine production was not found in supernatants, confirming the effective cytokine blockage. Cells were stained with CD45-PerCP-Cy5.5 (2D1) (eBioscience, San Diego, CA, USA), CD91-FITC (A2-MR-α2), CD14-PE-Cy7 (M5E2) and CD16-A700 (3G8) (BD Biosciences) to discriminate between monocyte subsets, as detailed in the standardized flow cytometry assay [35]. After cell membrane permeabilization using BD Intrasure permeabilizing solution (BD Biosciences), intracellular staining was performed to detect proinflammatory cytokines with the following antibodies: IL-1β-Α647 (JK1B-1) (BioLegend, San Diego, CA, USA), which recognized both pro- and active isoforms of IL-1β, IL-6-BV421 (MQ2-13A5), MCP-1-PE (5D3-F7) (BD Biosciences) and TNFα-BV711 (Mab11) (BioLegend). Cell samples were analyzed using the BD SORP LSRII and data analyzed using BD FACSDiva software (BD Biosciences).

### DNA extraction and real-time PCR analysis

Enriched blood monocytes and neutrophils were resuspended in TRIzol Reagent (Life Technologies, Burlington, ON, Canada) and DNA extraction was performed according to the manufacturer’s instructions. DNA was amplified using GoTaq qPCR MasterMix (Promega, Madison, WI, USA) on a Rotor Gene 3000 (Montreal Biotech Inc., Montreal, QC, Canada). Purified EBV was obtained from EBV B-cell lineage B95.8 as described [30, 33]. EBV dsDNA was isolated and purified as reported [30]. The following primers were used to detect BamHI-W EBV gene [29] (NCBI reference sequence NC_007605, Human herpes virus 4 type 1 complete genome, http://www.ncbi.nlm.nih.gov/nuccore/82503188) (forward: 5′-GCG CCA TTT TGT CCC CAC GC-3′ and reverse: 5′-TGG CCT AGC AAC GCG AAC CC-3′) and human glyceraldehyde-3-phosphate-deshydrogenase (hGAPDH) gene (forward: 5′-ATG CTG CAT TCG CCC TCT TAA TGG-3′ and reverse: 5′-AGG CGC CCA ATA CGA CCA AAT CTA-3′). Purified DNA from EBV-positive B95.8 B-cell line (Advanced Biotechnologies Inc., Columbia, MD, USA) was used as positive control for BamHI-W amplification.

### Statistical analysis

Nonparametric one-way analysis of variance (Kruskal–Wallis test) followed by Dunn’s post-hoc test were used to compare multiple groups. The nonparametric Student’s *t* test (Mann–Whitney) (unpaired, two-tailed) was performed for two experimental groups. Analyses were performed using GraphPad Prism version 6.00 for Windows (GraphPad Software, La Jolla, CA, USA).

## Results

### CD14^+^ CD16^+^ and CD14^low^ CD16^++^ monocyte levels are increased in blood from patients with active RA

**Fig. 1:**
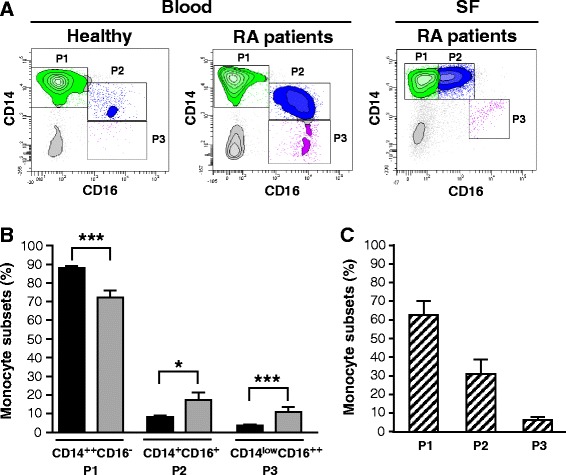
Levels of monocyte subsets in blood and synovial fluids from rheumatoid arthritis (*RA*) patients. **a** Flow cytometry analysis of CD14^++^ CD16^−^ (P1), CD14^+^ CD16^+^ (P2) and CD14^low^ CD16^++^ (P3) blood monocytes from healthy controls and RA patients and of synovial monocytes from RA patients. Data are cell populations of representative donors. **b** Percentage of blood monocyte subsets from healthy controls and RA patients. *Black bars* healthy donors, *gray bars* RA patients. **c** Percentage of monocyte subsets from synovial fluid of RA patients. Data represent the mean ± standard error of the mean. *Striped bars* RA patient synovial fluids (*SF*): *p ≤0.05 and ***p ≤0.001

Three distinct monocyte subsets are now defined in humans, two of them expressing the CD16 marker. While classical monocytes were suggested to play a significant role in the progression of several diseases, the trafficking of separately defined intermediate (CD14^+^ CD16^+^) and nonclassical (CD14^low^ CD16^++^) monocytes during RA was not fully investigated. We have first determined by flow cytometry the proportion of these three monocyte subsets in blood of patients with active RA compared to healthy volunteers. Monocyte subsets isolated from blood and synovial fluids of RA patients were gated as P1 (CD14^++^ CD16^−^), P2 (CD14^+^ CD16^+^) and P3 (CD14^low^ CD16^++^) (Fig. 1a). Gating of P2 and P3 subsets from SF of RA patients was found to slightly differ from blood cell gating, presumably due to their morphology and respective environment. Compared to healthy volunteers, we observed a reduced proportion of CD14^++^ CD16^−^ in blood from RA patients and a significant increase of both CD14^+^ CD16^+^ and CD14^low^ CD16^++^ monocytes (Fig. 1a–b). These results are in line with previous reports showing that the whole blood CD16^+^ monocyte population increases in patients with active RA [10, 36]. We have also evaluated the proportion of monocyte subsets that migrate into the joints of RA patients. In patients (n = 7) with knee joint effusion, we detected an elevated proportion of CD14^++^ CD16^−^ and CD14^+^ CD16^+^ monocytes but a very low number of CD14^low^ CD16^++^ monocytes (Fig. 1a, c). These results indicate that while all subsets of monocytes are detected in SF from RA patients, the classical CD14^++^ CD16^−^ and the intermediate CD14^+^ CD16^+^ monocytes appear to be predominantly recruited in the joints.

### Monocyte subsets from patients with active RA express increased levels of TLR2 and TLR9

**Fig. 2:**
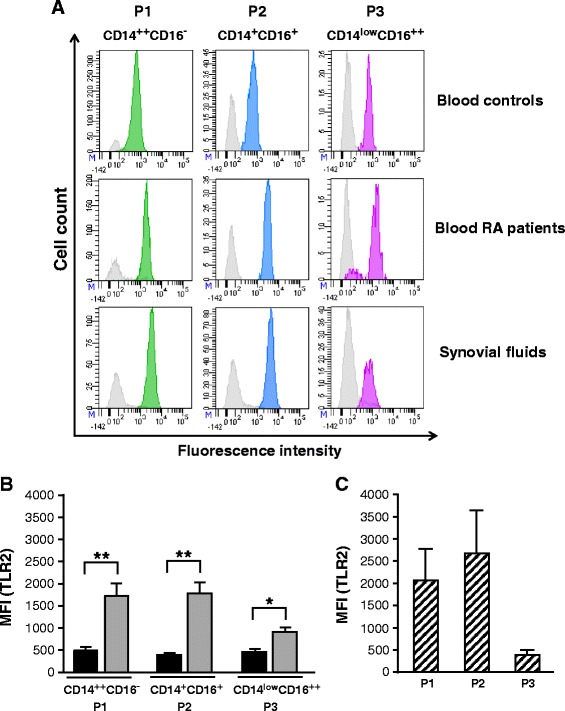
Toll-like receptor2 (*TLR2*) expression levels are increased in blood and synovial monocyte subsets of rheumatoid arthritis (*RA*) patients. **a** Flow cytometry analysis of TLR2 expression on blood monocyte subsets from healthy controls and RA patients. TLR2 expression for synovial monocyte subsets is also shown for RA patients. TLR2-positive cells appear in *green* (*P1*), *blue* (*P2*) or *purple* (*P3*). *Gray histograms* represent internal negative controls. Data are cell populations of representative donors. **b** TLR2 expression on each blood monocyte subset of healthy controls and RA patients is presented as mean fluorescence intensity (*MFI*) (mean ± standard error of the mean (SEM)). *Black bars* healthy donors, *gray bars* RA patients. **c** TLR2 expression in the monocyte subsets from synovial fluids of RA patients is presented as MFI (mean ± SEM). *Striped bars* RA patient synovial fluids: *p ≤0.05 and **p ≤0.01

**Fig. 3:**
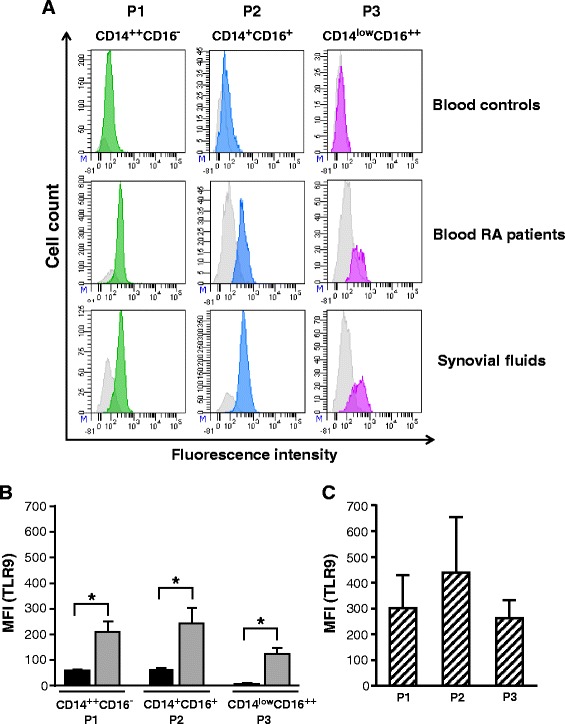
Increased level of toll-like receptor 9 (*TLR9*) on circulating and synovial monocyte subsets of rheumatoid arthritis (*RA*) patients. **a** Flow cytometry analysis of TLR9 expression on blood monocyte subsets from healthy controls and RA patients. TLR9 expression for synovial monocyte subsets is also shown for RA patients. TLR9 expression appears in *green* (P1), *blue* (P2) or *purple* (P3). *Gray histograms* represent internal negative controls. Data are cell populations of representative donors. **b** TLR9 expression on blood monocyte subsets from healthy controls and RA patients are presented as mean fluorescence intensity (*MFI*) (mean ± standard error of the mean (SEM)). *Black bars* healthy donors, *gray bars* RA patients. **c** TLR9 expression in the monocyte subsets of RA patient synovial fluids is presented as MFI (mean ± SEM). *Striped bars* RA patient synovial fluids: *p ≤0.05

Monocytes are known to drive inflammation in tissues where they are recruited. They can recognize various ligands through TLRs, a process leading to the production of several proinflammatory mediators. Although enhanced expression of TLR2 and TLR9 was demonstrated in the synovium of active RA patients, their distribution on all three monocyte subsets remains to be investigated. We first determined the expression levels of TLR2 and TLR9 on all three blood monocyte subsets isolated from RA patients and healthy controls. Staining of blood monocyte subsets with anti-TLR2 monoclonal antibody clearly showed increased fluorescence intensity levels of TLR2 on all three subsets of monocytes of patients with active RA compared to monocytes of healthy controls (Fig. 2a–b). When looking at TLR2 intensity levels on monocytes isolated from synovial fluids, expression levels were also detected on all three subpopulations of monocytes but found particularly high on CD14^++^ CD16^−^ and CD14^+^ CD16^+^ monocytes (Fig. 2a, c). The intensity levels of TLR9 were significantly increased on all three monocyte subsets (Fig. 3a–b). Furthermore, TLR9 expression profiles were also enhanced on monocyte subpopulations isolated from synovial fluids of patients with active RA (Fig. 3a, c). These data suggest that expression of TLR9 is regulated during active RA and that TLR9 could play a significant role as an innate sensor contributing to sustained inflammation in the joints of patients with active RA.

### Increased expression of TLR2 and TLR9 on neutrophils of RA patients

**Fig. 4:**
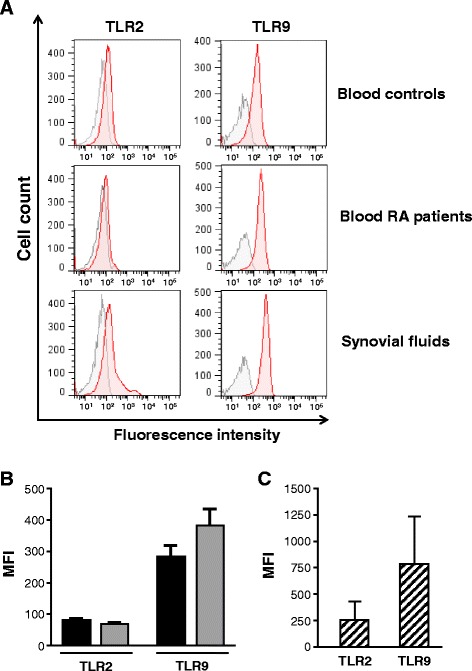
Expression of toll-like receptor 2 (*TLR2*) and *TLR*9 in neutrophils of rheumatoid arthritis (*RA*) patients. **a** Flow cytometry analysis of TLR2 and TLR9 expression in neutrophils isolated from blood of healthy controls and RA patients and synovial fluids of RA patients. TLR2 and TLR9 expression is represented by *red histograms* whereas negative control is represented by *gray histograms*. Data are neutrophils of representative donors. **b** TLR2 and TLR9 expression on blood neutrophils from healthy controls and RA patients are presented as mean fluorescence intensity (*MFI*) (mean ± standard error of the mean (SEM)). *Black bars* healthy donors, *gray bars* RA patients . **c** TLR2 and TLR9 expression on synovial fluid neutrophils from RA patients are presented as MFI (mean ± SEM). *Striped bars* RA patient synovial fluids

As neutrophils express several TLRs including TLR2 and TLR9, we wanted to determine as we did for monocytes, whether the expression of these TLRs is also increased on neutrophils isolated from blood and synovial fluids from RA patients. The fluorescence intensity level of TLR2 was not significantly affected on blood neutrophils from RA patients and remained comparable to blood neutrophils obtained from healthy controls (Fig. 4a–b) which express low levels of TLR2 [37, 38]. Cytometry analysis of RA blood neutrophils also demonstrated modest expression of TLR9, which was similar to neutrophils obtained from healthy volunteers (Fig. 4a–b). In contrast, TLR2 and TLR9 expression profiles on neutrophils isolated from synovial fluids were quite different (Fig. 4a, c). Indeed we observed that both TLR2 and TLR9 expression levels were significantly increased in synovial neutrophils. While these results cannot be compared to healthy controls for obvious reasons, TLR2 and TLR9 levels on synovial neutrophils were still higher than blood neutrophils isolated from either healthy controls or RA patients. We thus reasoned that such increased expression of TLR2 and TLR9 might reflect a more sustained activation of neutrophils induced by the release of TLR2/TLR9 agonists inherent to the RA synovial environment. Taken together, these results could suggest that following their migration into the joints, neutrophils may present enhanced responsiveness to TLR2 and TLR9 ligands.

### Enhanced responsiveness of monocyte subsets of patients with active RA to synthetic and viral TLR2 and TLR9 agonists

Increased expression levels of TLR2 and TLR9 in monocytes from RA patients may indicate that these cells are potentially more sensitive to TLR2 and TLR9 ligand stimulation. We next wanted to determine whether increased expression of TLR2 and TLR9 on different monocyte subsets could also correlate with enhanced production of inflammatory cytokines when stimulated with TLR2 and TLR9 ligands. To evaluate which monocyte population contributes to cytokine production in RA patients, isolated mononuclear cell populations of RA patients and healthy controls were stimulated in vitro with synthetic and viral TLR2 agonists, LTA and EBV particles [33] and with TLR9 agonists, CpG and EBV DNA [30]. Viral infection has for a long time been proposed to contribute to exacerbate RA symptoms in susceptible patients. We chose EBV components as viral agonists because this virus is recognized by both TLR2 and TLR9 [30, 33] and because the recruited RA patients had abnormal elevated anti-EBV titers. After 5 hours of stimulation, ICCS was performed on IL-1β, IL-6, TNFα and MCP-1 on the three distinct monocyte subsets and expression levels was monitored by flow cytometry. This approach was chosen over sorted monocyte populations because it would be technically difficult to purify each monocyte subset with the small blood samples obtained from RA patients.

**Fig. 5:**
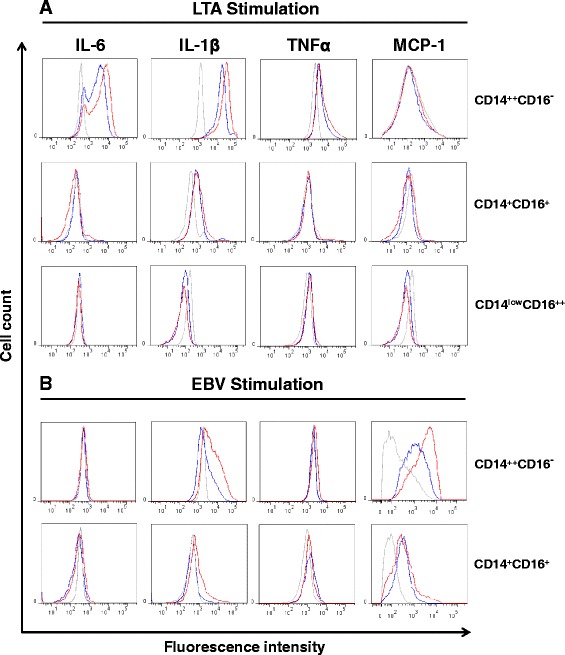
Intracellular cytokine expression in monocyte subsets of rheumatoid arthritis (RA) patients after stimulation with toll-like receptor (TLR2) agonists. Mononuclear cells from RA patients and healthy donors were enriched and stimulated with lipoteichoic acid (*LTA*) (**a**) and Epstein–Barr virus (*EBV*) particles (TLR2 ligands) (**b**) in vitro. After a 5-hour stimulation, the stimulated cells were harvested and cytokine expression levels were assessed by intracellular flow cytometry. Briefly, cells were stained with CD45, CD91, CD14 and CD16, permeabilized and intracellular staining was performed on proinflammatory cytokines: IL-6, IL-1β, TNFα and monocyte chemoattractant protein-1 (*MCP-1*). Intracellular cytokine expression is shown for classical CD14^++^ CD16^−^, intermediate CD14^+^ CD16^+^ and nonclassical CD14^low^ CD16^++^ monocyte subsets. *Gray* non-stimulated samples, *Blue* healthy donors, *red* RA patients. *Representative cytometry histograms* are representative of ten donors

Stimulation of RA monocytes with the synthetic TLR2 agonist, LTA, induced an increase of IL-6, IL-1β and TNFα expression in CD14^++^ CD16^−^ subsets when compared to healthy donors (Fig. 5a). IL-1β expression was also increased in the CD14^+^ CD16^+^ subset upon LTA stimulation, but levels were equivalent in both healthy and RA patients. LTA treatment did not induce the synthesis of monocyte chemoattractant protein-1 (MCP-1) in any subpopulation of monocytes. Interestingly, RA patient monocytes responded distinctly to the second TLR2 agonist, EBV virions. Indeed, intracellular expression IL-1β and MCP-1 was increased in both CD14^++^ CD16^−^ and CD14^+^ CD16^+^ subsets of RA patients when compared to healthy donors (Fig. 5b). This result is consistent with findings that show that EBV binding to monocytes leads to MCP-1 production [33]. Our observations show that EBV virions also lead to an increase in the production of IL-1β in CD14^++^ CD16^−^ and CD14^+^ CD16^+^ in arthritic patients (Fig. 5b). This result entails that IL-1β expression is probably mediated by envelope protein recognition by surface TLRs, most likely TLR2. We must also consider that the IL-1β gene could be activated following viral entry into cells [39].

Interestingly, our results show that healthy and RA patients did not produce TNFα in response to EBV. This is consistent with results that demonstrated that infectious EBV strongly inhibit TNFα in primary mononuclear cells and in myelo-monocytic cell lines [40]. Furthermore, we have observed that EBV stimulation appeared to have completely eliminated the CD14^low^ CD16^++^ monocytes, as witnessed by flow cytometry. The basis for this selective depletion of CD14^low^ CD16^++^ monocyte is unknown, but it was unmistakable for both healthy and RA samples. In all, these results suggest that classical CD14^++^ CD16^−^ and CD14^+^ CD16^+^ monocyte subsets have been sensitized and can participate more robustly to the proinflammatory cytokines response when stimulated with TLR2 agonist, including EBV particles.

**Fig. 6:**
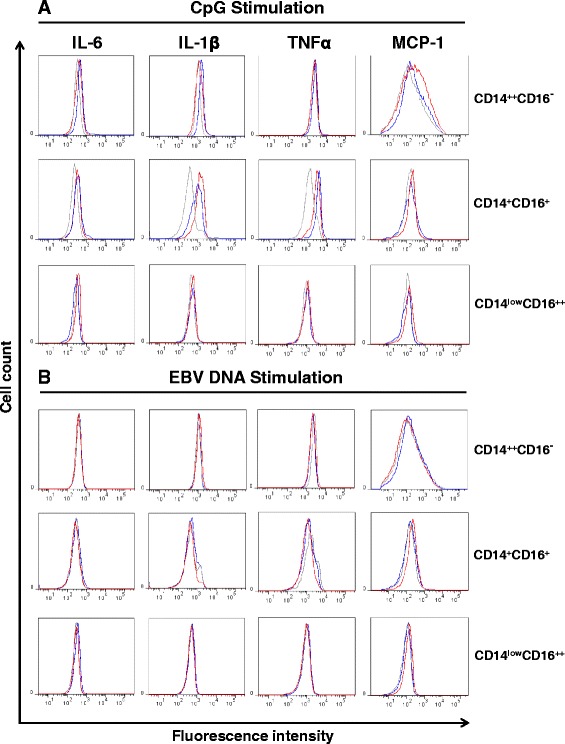
Intracellular cytokine expression in monocyte subsets from rheumatoid arthritis (RA) patients following toll-like receptor 9 (TLR9) stimulation. Mononuclear cells from RA patients and healthy donors were enriched and stimulated with CpG-2006 (**a**) and Epstein–Barr virus (*EBV*) DNA (TLR9 ligands) (**b**) in vitro. Gating strategy is described in Fig. 5. Intracellular cytokine level for IL-6, IL-1β, TNFα and monocyte chemoattractant protein-1 (*MCP-1*) are shown for all three subsets of monocytes. *Gray* non-stimulated samples, *Blue* healthy donors, *red* RA patients. *Representative cytometry histograms* are representative of ten donors

Stimulation with TLR9 agonist, CpG, induced a heightened intracellular expression of both IL-1β and TNFα in CD14^+^ CD16^+^ monocyte subsets, where their respective cytokine expression was similar for both healthy and RA patients (Fig. 6a). We also observed a significant increase in MCP-1 production in the CD14^++^ CD16^−^ monocyte population from RA patients. In the current settings, stimulation with EBV DNA did not appear to induce a broad proinflammatory cytokine response in the respective monocyte subset (Fig. 6b).

Together, these results show that monocytes from RA patients display a heightened inflammatory response state, as illustrated by increased production of proinflammatory cytokines, when stimulated with TLR2 and TLR9 ligands. They also underline the importance of both monocyte subsets, CD14^++^ CD16^−^ and CD14^+^ CD16^+^, as producers of inflammatory mediators following recognition of TLR ligands. The presence of IL-6 and MCP-1 detected in synovial fluids from RA patients is in line with the expression profile measured by intracellular staining (Table 2).

**Table 2:** Cytokine detection in plasma and synovial fluids of RA patients

	Blood	Synovial fluids
IL-6	23.6 ± 5.3	11484.5 ± 3960.2
IL-1β	N.D.	4.0 ± 1.6
TNF α	N.D.	2.8 ± 0.4
MCP-1	87.2 ± 11.1	975.4 ± 691.9

Concentrations (pg/ml) of cytokines (IL-6, IL-1β, TNFα, monocyte chemoattractant protein-1 (*MCP-1*) were monitored by cytometric bead array in plasma and synovial fluids from rheumatoid arthritis (*RA*) patients. Presence of cytokines was not detected in plasma from healthy volunteers. Data are mean ± standard error of the mean. *N.D.* not detected

### EBV genome is present in monocytes and neutrophils of patients with active RA

For many years, EBV has been suspected to contribute to the exacerbation of inflammation in susceptible patients suffering from auto-immune diseases like RA. Because we have observed that monocytes of patients with active RA have enhanced responsiveness to EBV, this prompted us to evaluate whether circulating monocytes and neutrophils of RA patients harbor the EBV genome. First, we measured elevated levels of anti-EBV antibodies in sera from RA patients compared to the EBV-seropositive control group, suggesting that viral reactivation occurs in these patients (data not shown). Cellular DNA was next extracted from enriched blood monocytes and neutrophils from RA patients and from seropositive healthy volunteers and the presence of the EBV genome was evaluated by real-time PCR analysis of the BamHI-W repeated sequence of the viral genome as we previously reported [29]. The presence of the EBV genome was detected in circulating monocytes from eleven RA patients tested and in blood neutrophils from five of these patients (Table 3). Synovial monocytes from three patients were found positive for the presence of the EBV genome, one of them also having EBV-positive neutrophils, suggesting that both monocytes and neutrophils could contribute to the transport of the virus into the joints of those patients.

**Table 3:** Detection of the Epstein–Barr virus (EBV) genome in monocytes and neutrophils from rheumatoid arthritis (RA) patients

	Blood		Synovial fluids
Cell populations	Healthy controls	RA patients	RA patients
Monocytes	1/12	11/23	3/7
Neutrophils	0/12	5/23	1/7

DNA was isolated from monocytes and neutrophils and analyzed by real-time PCR for the presence of BamH1-W sequence in the EBV genome as detailed in “Methods”. Results are presented as number of positive samples over all donors tested

## Discussion

Some patients with active RA have fluctuations of disease symptoms and transient flare-up episodes during treatment, suggesting intermittent activation of cells or immune pathways involved in driving inflammation. Circadian and circannual biological rhythms are known to influence the pathophysiology and clinical symptoms of RA [41]. Clinical symptoms and disease activity are influenced by seasonal changes [42] through the alternation of sunlight and darkness. For example, the low levels of vitamin D in winter and spring are associated with increased risks of disease onset and severity of disease activity and outcomes [43]. Vitamin D regulates both innate and adaptive immunity, potentiating the innate response but suppressing adaptive immunity [44]. Its synthesis is increased in monocytes/macrophages in the presence of bacterial and viral infections, a phenomenon that contributes to initiate innate defense against pathogens, including viruses. The onset and severity of RA might also be due to the seasonality of infections.

Previous studies have indicated that monocytes expressing a CD16^+^ phenotype are expanded in the blood of patients with inflammatory diseases like RA, making these cell populations a plausible marker of disease activity [8, 45]. However, those studies have investigated CD16^+^ monocytes as a unique population, thus masking the reliable functions of each CD16^+^ subset. In the present study, we have separately defined all three subsets of monocytes in order to evaluate their individual role in active RA. We have observed a significant increase of both subsets of CD16^+^ monocytes (CD14^+^ CD16^+^, CD14^low^ CD16^++^) in blood from patients with active RA. On the other hand, the decrease of classical blood monocytes (CD14^++^ CD16^−^) could reflect a constant recruitment of these cells to the inflamed tissue in order to regulate local inflammation. The marked presence of classical and intermediate (CD14^+^ CD16^+^) monocytes in synovial fluids from patients presenting with knee effusion emphasize the role played by these two monocyte subsets in the inflamed tissues of RA patients.

The increased expression of CD14^+^ CD16^+^ and CD14^low^ CD16^++^ monocyte subsets in patients with active RA may suggest the progression of the disease [10, 46, 47], but may also reflect the activation of the monocyte population. This hypothesis is in line with the expression levels of TLR2 and TLR9 detected on individual monocyte subsets. In fact, staining for TLR2 revealed a marked increased expression of TLR2 on blood and synovial CD14^++^ CD16^−^ and CD14^+^ CD16^+^ monocytes from RA patients and to a lesser extent on blood CD14^low^ CD16^++^ monocytes. We also detected an enhanced expression of TLR9 on all three subsets of monocytes regardless of their origin (blood or synovial fluids). While the exact roles of TLR2 and TLR9 in RA remain speculative, it has, however, been suggested that the presence of TLR2 in synovial tissue and macrophages of patients with clinically active disease contribute to symptom severity via the production of inflammatory cytokines [18, 20]. Another important issue is that synovial fibroblasts from RA patients release chemokines in response to TLR2 triggering [48]. The presence of TLR2 on CD16^+^ blood monocytes is also proposed to contribute to the production of TNFα in RA patients [17]. Therefore, the enhanced expression of TLR2 that we detected on monocytes from RA patients may indeed contribute to the secretion of various mediators, including chemotactic factors involved in the recruitment of blood cells into the joint.

Little is known about the role of TLR9 in RA. However, the therapeutic effect of hydroxychloroquine, an inhibitor of TLR9 signaling, suggests that TLR9 might participate in the severity of inflammatory symptoms in RA [49]. We found that all blood and synovial monocyte subsets, particularly CD14^++^ CD16^−^ and CD14^+^ CD16^+^ monocytes, express significant levels of TLR9, which highlights a potential contribution of this TLR in RA. Interestingly, we also detected a marked increase of TLR9 on synovial neutrophils from RA patients. Neutrophils being essential to control invading agents and eliminate cell debris, such increased expression of TLR9 in neutrophils may thus suggest that they are potentially more susceptible to producing inflammatory mediators following their migration into the joint.

The higher levels of TLR2 and TLR9 detected on different monocyte subsets from RA patients were in line with the production of cytokines by these cells in response to stimulation with TLR2 and TLR9 ligands. Indeed, both TLR2 agonists tested were found to induce an increase in proinflammatory cytokine production in cells isolated from RA patients compared to healthy controls. To refine the functional characterization and to identify the monocyte subsets that produce these cytokines, we performed intracellular measurement in each RA subset of monocytes. Our results clearly show that proinflammatory cytokines were found mostly in CD14^++^ CD16^−^ and CD14^+^ CD16^+^ monocyte subsets confirming the importance of these subsets in the TLR2-dependent proinflammatory events. Interestingly, our results also indicate that both TLR2 agonists induce a distinct immune response cytokine profile. On the one hand, LTA triggers the production of IL-1β in both CD14^++^ CD16^−^ and CD14^+^ CD16^+^ monocytes, while production of IL-6 and TNFα is restricted to CD14^++^ CD16^−^ monocytes. Likewise, EBV virions also induce clear proinflammatory cytokine secretion profiles with increased levels of IL-1β and MCP-1 by both CD14^++^ CD16^−^ and CD14^+^ CD16^+^ monocytes.

The distinct cytokine profiles of monocyte subsets in response to LTA stimulation presume that these cells should also be sensitive to many other endogenous TLR2 agonists released in inflamed joints and also that regulation of cytokine genes may differ from one subtype of monocytes to another. The massive detection of intracellular MCP-1 in two RA monocyte subsets following EBV stimulation suggests that these cells could potentially trigger the release of MCP-1, a potent chemokine involved in the recruitment of inflammatory monocytes, once they are in the synovial environment. As expected, EBV stimulation did not induce the activation of the TNF gene, being recognized as a suppressor of TNF synthesis [40, 50].

Stimulation with the TLR9 agonist CpG showed a distinct intracellular cytokine profile compared to the stimulation with TLR2 agonists. Indeed, production of IL-1β and TNFα was mainly detected in CD14^+^ CD16^+^ monocytes, while modest production of MCP-1 was detected in CD14^++^ CD16^−^ monocytes. EBV DNA treatment failed to accumulate detectable levels of intracellular cytokines, confirming that in fact, the cytokines tested are mainly regulated through the triggering of TLR2. Unfortunately, no significant change in cytokine profiles were observed in CD14^low^ CD16^++^ monocyte but this might reflect the limitation of the experimental approach where very low numbers of these cells are isolated from patient tissues.

The enhanced secretion of cytokines by monocyte subsets stimulated with EBV virions might reflect the susceptibility of these cells to viral components. In this regard, we detected the presence of EBV genome in monocytes from a large proportion of patients with active RA. Neutrophils from five of those RA patients were also found to contain EBV genome, indicating that both these cell populations may contribute to the dissemination of viral particles in patients with active RA, as previously suggested [28, 29]. Therefore, as EBV is recognized by TLR2 and TLR9 and can also enhance TLR9 expression following its entry into monocytes [30, 33], triggering of these sensors by released viral particles may thus contribute to sustained cellular activation and abnormal production of inflammatory mediators. Our observations also reinforce the link that infectious agents may lead to exacerbation of arthritis and disease flares in susceptible patients. Indeed, despite good adherence to disease-modifying antirheumatic drugs (DMARDs), such as methotrexate and hydroxychloroquine, some patients have severe intermittent fluctuations in the clinical activity of the disease.

In light of our observations, we suggest that both monocyte subsets, e.g., CD14^++^ CD16^−^ classical and CD14^+^ CD16^+^ intermediate monocytes play an active role in the severity of inflammation in active RA and that CD14^++^ CD16^−^ classical monocytes could also contribute to amplify the inflammation by secreting proinflammatory cytokines and potent chemoattractants. We assume that levels of both TLR2 and TLR9 are appealing markers in determining the level of activation of monocytes. It is thus plausible to consider a scenario that may engage a sequence of at least two TLRs: TLR2 may generate the first signal following recognition of self molecules released from damaged cells, and TLR9 creates a feedback loop by binding DNA motifs internalized by phagocytes like neutrophils and macrophages. We presume that TLR2 and TLR9 can also be activated by viral components (like EBV) in the synovial compartment, a mechanism causing exacerbation of arthritis in susceptible RA patients. The combined activation of TLR2 and TLR9 may thus contribute to sustain inflammation in the joints of RA patients by inducing a wide range of chemokines and cytokines. In the absence of efficient mechanisms that can regulate such an amplification loop, an excessive production of inflammatory mediators may result in increased severity of RA symptoms. Overall, our results in RA patients with a high disease activity score highlight the combined potential of TLR2 and TLR9 in the pathophysiology of RA and suggest the possible involvement of an opportunistic pathogen like EBV in the exacerbation of RA symptoms.

## Conclusions

Results presented in this study demonstrate that blood and synovial monocyte subsets isolated from patients with active RA have high levels of TLR2 and TLR9 and increased production of inflammatory cytokines in response to synthetic and viral TLR2 and TLR9 agonists. Such mechanisms may thus contribute to exacerbation of arthritis in patients with active RA.

## Abbreviations

CRP: C-reactive protein, DAS28, disease activity score in 28 joints
EBV: Epstein–Barr virus
ELISA: enzyme-linked immunosorbent assay
ESR: erythrocyte sedimentation rate
FITC: fluorescein isothiocyanate
ICCS: intracellular cytokine staining
IL: interleukin
LTA: lipoteichoic acid
MCP-1: monocyte chemoattractant protein-1
MFI: mean fluorescence intensity
PBMC: peripheral blood mononuclear cells
RA: rheumatoid arthritis
SF: synovial fluids
TLR: toll-like receptor
TNF: tumor necrosis factor
